# Acoustic Emission Characteristics and Damage Mechanisms Investigation of Basalt Fiber Concrete with Recycled Aggregate

**DOI:** 10.3390/ma13184009

**Published:** 2020-09-10

**Authors:** Guodong Li, Li Zhang, Fengnian Zhao, Jiaqi Tang

**Affiliations:** 1 Transportation Institute, Inner Mongolia University, Hohhot 010070, China; tension828@163.com (L.Z.); imuzhaofengnian@outlook.com (F.Z.); tangjiaqi423@163.com (J.T.); 2 Inner Mongolia Engineering Research Center of Testing and Strengthening for Bridges, Hohhot 010070, China

**Keywords:** basalt fiber, recycled concrete, acoustic emission, b-value, cluster analysis, RA-AF

## Abstract

This paper presents the compression failure process of basalt fiber concrete with recycled aggregate and analyzes the main factors of basalt fiber and recycled aggregate affecting the compressive strength of recycled concrete. The damage mechanism of recycled aggregate concrete is analyzed by the acoustic emission technique. With the method of acoustic emission (AE) b-value analysis, the evolution and failure process of recycled concrete from the initial defect microcrack formation to the macroscopic crack is studied. Based on the AE clustering analysis method, the damage state of recycled concrete under load grade is investigated. Finally, the failure mode of recycled concrete is explored according to the RA-AF correlation method. The results show that when the concrete reaches the curing age, the strength grade of basalt fiber regenerated coarse aggregate concrete is the highest. The basalt fiber increases the strength of regenerated fine concrete by 4.5% and the strength of coarse concrete by 5%, and reduces the strength of fully recycled aggregate concrete by 6.7%. The b-value divides concrete into three stages: initial damage, stable development of internal damage, and internal damage. The variation of AE energy, count, and event number is related to AE activity and crack growth rate. Matrix cracking is the main damage state of concrete, which is greatly affected by the strength of cement mortar. The load grade of fiber cracking in fully recycled aggregate, recycled fine aggregate, and recycled coarse aggregate concrete is 65, 90, and 85%, respectively. Basalt fiber increases the tensile failure event point of recycled concrete and delays the cracking of recycled concrete under compression. When the load grades of fully recycled fiber, recycled fine aggregate fiber, and recycled coarse aggregate fiber concrete are 65–95, 90–100, and 85–100%, respectively, the tensile failure activity increases.

## 1. Introduction

Since the reform and opening up, China has made remarkable achievements in infrastructure construction. However, about 600 million tons of construction waste is also generated in China every year, of which concrete waste accounts for 30–35% [1]. How to effectively reuse waste concrete has thus become an important issue. With the development of modern concrete technology, recycled concrete technology has been proposed and is gradually being applied to actual projects, which can effectively solve the problem of waste concrete treatment.

Although the failure mechanism of recycled aggregate is similar to that of natural aggregate, there are great differences in their physical properties. The density of recycled aggregate is low, and old mortar and microcracks are commonly adhered to the surface [2,3]. The limited shrinkage and wear resistance of recycled concrete limit its application in practical engineering [4,5,6]. Studies exploring the mechanical properties of some recycled coarse concrete and fine concrete have obtained different conclusions and failed to achieve a unified standard [7,8,9,10,11,12,13]. Some scholars adopted advanced mixing and chemical treatment methods to optimize the recycled aggregate, which improved the physical characteristics of the recycled aggregate and the durability of the recycled concrete [14,15]. It studied the conventional and equivalent volume (EMV) mortar mix proportion design method of steel fiber recycled aggregate concrete (RAC) and the effects of mixture ratio. The results showed that recycled aggregates and the existence of the steel fiber reinforced concrete increased the water-absorbing capacity by 49% over the natural concrete, and the tensile splitting strength of steel fiber recycled concrete was higher than ordinary concrete [16]. In addition, the effects of adding steel fiber to recycled aggregate concrete on ordinary concrete, self-compacting concrete, and high-performance concrete have recently been studied [17,18,19]. Compared with steel fiber, basalt fiber base material has no other excess impurities, and has high and low-temperature resistance, environmental protection, and low cost. Basalt fiber is widely used in recycled concrete due to its good acid and alkali resistance, corrosion resistance, high-temperature resistance, and other characteristics. The first researchers to put forward the mechanism of fiber reinforced concrete were J.P. Romualoli and B. Batson [20,21]. Many scholars have studied recycled concrete using the basalt fiber volume rate and the replacement rate of recycled coarse aggregate as the research variables [22,23,24,25,26,27]. It studied the fracture characteristics of high-performance concrete mixed with basalt fiber and polypropylene fiber through the method of concrete fracture performance testing [28]. Jiyang Wang used the eccentric compression test method and concluded that basalt fiber improved the bearing capacity and ductility of damaged masonry [29]. It studied the effect of water film thickness and fiber content on mortar strength and proposed a theoretical model of basalt fiber to reinforce mortar strength [30]. In recent literature reviews, research on chemical modified recycled carbon fiber composite, glass fiber reinforced concrete, and tremie reinforced concrete has been mentioned [31,32,33].

**Figure 1:**
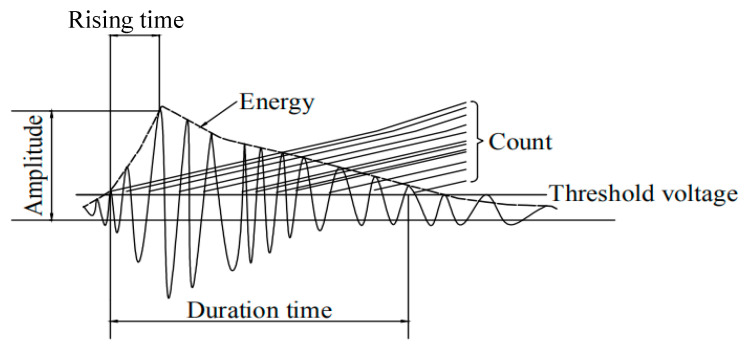
Principle of acoustic emission.

Acoustic emission (AE) technology has been adopted by many structural tests, and the test principle is shown in Figure 1. AE technology refers to the process of transmitting the local transient energy by elastic wave signal when the material has elastic or plastic deformation, and is also known as stress wave emission. Because the material has discrete and non-uniform characteristics, stress concentration can easily occur after the load is applied, resulting in strain, relative displacement, or microcracks in the material. This process generates local transient energy. When the AE signal detects the local part of the material to the damage source, the transient stress wave released by the signal is transmitted to the surface of the material, causing mechanical vibration. The AE probe converts the mechanical vibration signal into an electrical signal. After the electrical signal is processed by an amplifier, the AE characteristic parameters are recorded in the form of waveforms. According to the changing rule of the characteristic parameters of the AE signal, the material can identify internal damage, the material crack development process, and early warnings of material damage.

Abouhussien and Hassan used the AE location method to study the internal damage of concrete and revealed the importance of AE characteristic parameters to detect concrete construction [34]. Watanabe T, Nishibata S, Hashimoto C et al. evaluated the crack evolution mechanism of recycled concrete and ordinary concrete according to the change rule of IB value [35]. Liu et al. studied the damage evolution process of carbon fiber/epoxy composite materials and used the amplitude of the AE characteristic parameters to divide the material damage into four types: matrix cracking, interlayer separation, interface debonding, and fiber fracture [36]. The research of Aslan, Monti A, Gutkin R et al. showed that changes in amplitude and peak frequency were closely related to the damage mechanism of composite materials [37,38,39]. Nawal, Kishor et al. used the RA-AF correlation analysis method to evaluate the tensile shear failure characteristics of concrete beams [40].

Scholars and experts have done a lot of research on the AE characteristics of concrete and fiber reinforced concrete, and have achieved corresponding results. However, research into the damage mechanism of basalt fiber recycled concrete remains lacking. In this paper, based on the existing research, using AE non-destructive testing as the technical means, and the aggregate collocation method and basalt fiber as the research variables, the design ratio of one natural concrete and six kinds of recycled concrete is designed. According to b-value, cluster analysis, and RA-AF, the evolution mechanism, damage state, and failure mode of concrete cracks are studied in depth by using the test method of AE to detect the compressive concrete. It is expected to provide a theoretical basis and guidance for engineering practice. 

## 2. Experimental Materials and Methods

### 2.1. Experimental Material

The cementitious materials used in the test were P.O42.5 ordinary Portland cement and type I high calcium fly ash. Physical performance indexes are shown in Table 1 and Table 2. The natural fine aggregate was natural river sand with a particle size range of 0.15–4.75 mm and a fineness modulus of 2.8. The natural coarse aggregate was crushed granite gravel, with the particle size range of 4.75–26.5 mm. Recycled aggregate was waste concrete test block; recycled fine aggregate was crushed slag with the particle size range of 0.15–4.75 mm, sand rate of 3.6; recycled coarse aggregate was crushed gravel with a particle size range of 4.75–26.5 mm. The apparent density of recycled coarse aggregate and fine aggregate was 2462 kg/m^3^ and 2401 kg/m^3^, the bulk density was 1484 kg/m^3^ and 1630 kg/m^3^, and the porosity was 46.8% and 43.6%, respectively. The water absorption rate of recycled coarse aggregate was 1.89%, the mud content of recycled fine aggregate was 6.22%, and the volume ratio of chopped basalt fiber was 0.1%. The concrete water-cement ratio was 0.3, and the design strength was C50. The curing temperature was 20 ± 2 °C, the humidity was more than 95%, and the curing period was 28 days. The physical performance indexes are shown in Table 3.

### 2.2. Concrete Mix Design

In the test, the proportion of materials in each part of the cement mortar and the continuous grading of coarse aggregate were kept unchanged, and the aggregate mix method and basalt were taken as the research variables. One kind of natural concrete mix proportion and six kinds of recycled aggregate concrete mix proportions were designed. In the reference concrete, the coarse and fine aggregates were natural aggregates, and the test number was recorded as J1. In fully recycled concrete and fiber fully recycled concrete, the aggregate was all recycled aggregate, and the test number was recorded as J2, J3. In the recycled/fiber recycled fine aggregate concrete, the fine aggregate was crushed slag, the coarse aggregate was natural, and the test number was recorded as J4, J5. In the recycled/fiber recycled coarse aggregate concrete, the coarse aggregate was recycled coarse aggregate, the fine aggregate was natural river sand, and the test number was recorded as J6, J7. The concrete mix design is shown in Table 4, and the specific aggregates are shown in Table 5.

### 2.3. Experimental Procedure and Method

**Figure 2:**
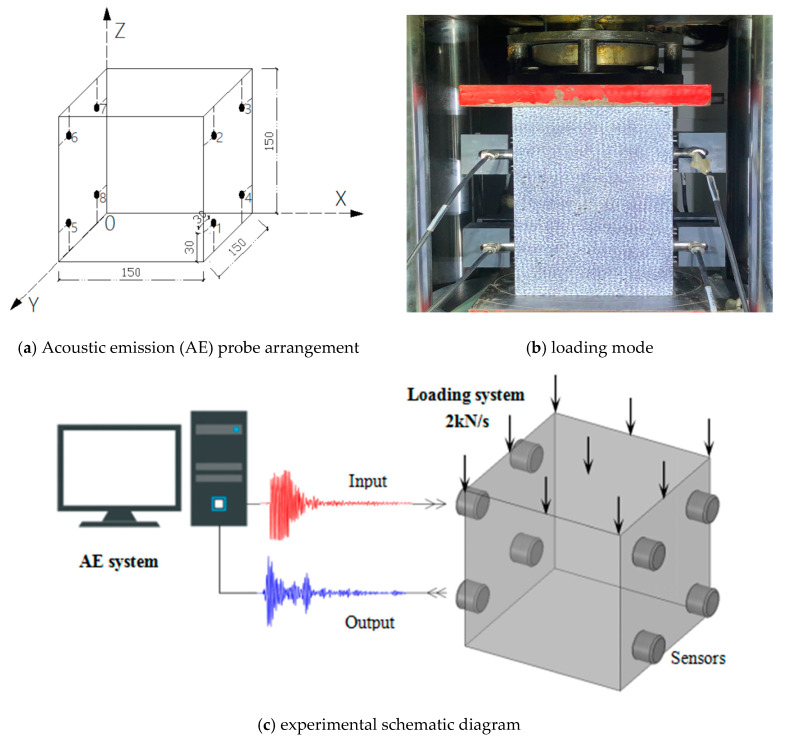
Acoustic emission (AE) probe arrangement and experimental schematic diagram. (Unit: mm).

The size of the concrete test piece was 150 mm × 150 mm × 150 mm. Three parallel test pieces were poured in each proportion. The test started when the curing age of the test piece reached 28 days. The AE test instrument was a Micro-II-Express 8-channel AE system and a R15I-AST longitudinal wave probe produced by Physical Acoustics of the United States. The probe size was 30 mmOD × 30 mmH, and AE real-time monitoring was maintained during the whole loading process. The arrangement of the AE probe and an experimental diagram is shown in Figure 2. The distance between each AE sensor and the edge of the test piece was 30 mm. The press was a TYA-2000, with loading speed of 2 kN/s and a preloading of 5 kN.

## 3. Results and Discussion

### 3.1. Compressive Strength

The factors influencing the compressive strength of concrete at 3, 7, and 28 days are discussed in Table 6. In the proportion of natural aggregate, the compressive strength of concrete reduced by 12.7, 9.9, and 4.1%, respectively, at 3, 7, and 28 days after using recycled coarse aggregate to replace natural coarse aggregate completely; in the proportion of fully recycled aggregate, the compressive strength of concrete decreased by 13.6, 12.6, and 8.5% in 3, 7, and 28 days, respectively, after using natural coarse aggregate to replace recycled coarse aggregate completely. In natural concrete, the compressive strength of concrete was reduced by 6.9, 8.4, and 8.9% at 3, 7, and 28 days after the natural river sand was completely replaced by crushed slag; in fully recycled aggregate concrete, the compressive strength of concrete was 18.9, 14.1, and 3.6% lower in 3, 7, and 28 days, respectively, after the natural river sand was completely replaced by crushed slag. The results show that compressive strength is related to the collocation method between aggregates. Aggregate is the same type of concrete, which can more easily form a stable aggregate mortar interface and has better compression characteristics.

According to the results of the table, when the concrete age is 3, 7, and 28 days, the strength of fully recycled concrete is reduced by 8, 9.2, and 6.7%, the strength of recycled fine aggregate concrete is increased by 1.9, 2.7, and 4.5%, and the strength of recycled coarse aggregate concrete is increased by 5.4, 1.7, and 5.0%. Basalt fiber improves the compressive strength of recycled fine and coarse aggregate concrete, but fails to improve the strength grade of fully recycled concrete. The reason is, in the fully recycled concrete, the water absorption of coarse and fine aggregate is large, and the sand ratio of fine aggregate is also relatively large. The cement paste content of the concrete is also lower than the other two recycled concrete proportions, and the fiber is wrapped by the cement paste to improve the mortar interface effect of the concrete, which leads to the concrete strength grade being reduced by the concrete mixed with basalt fiber. In conclusion, the best ratio of strength grade is J1, J2, and J7.

### 3.2. Stress-Strain Analysis of Concrete

**Figure 3:**
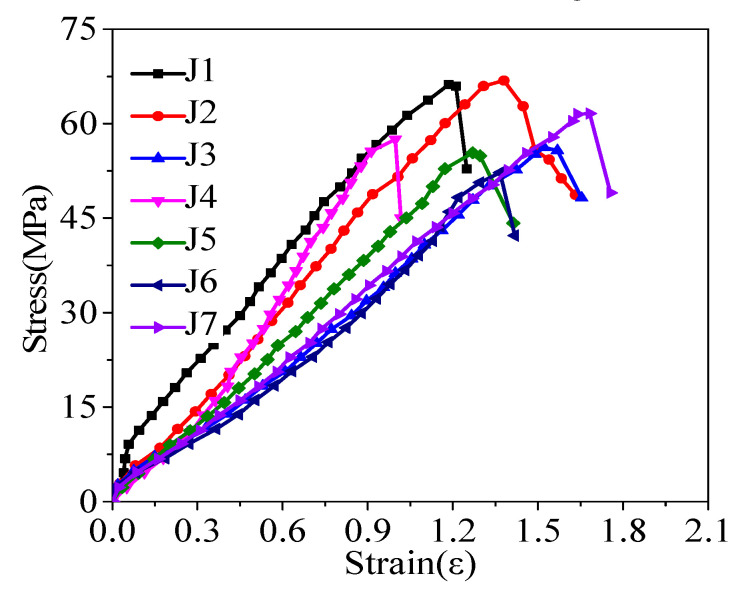
Stress-strain analysis of concrete.

The stress-strain curve of concrete is shown in Figure 3. According to the diagram, the stress-strain curve of concrete can be divided into the linear elastic stage and failure decline stage.

Compared with ordinary strength concrete, the stress-strain curve of concrete has some differences because the higher the concrete strength grade, the longer the linear elastic characteristics, high strength mortar, and aggregates, which affect the concrete bondability, compactness, and create fewer microcracks. The final damage is often aggregate damage, due to brittleness, and is the most obvious steep decline period. The second reason is that this test adopts the stress loading method as the ordinary testing machine. When the concrete reaches the axial compressive strength *f*_c_, the elastic strain energy gathered in the testing machine is greater than the strain energy absorbed by the specimen, which leads to the sudden destruction of the specimen, and the descending section of the measured specimen is not obvious. In this test, the concrete has high compressive strength and good compactness performance, so in the measured stress-strain curve of concrete, the linear elastic stage is relatively long, while the failure and failure decline stage is relatively short and steep.

### 3.3. Crack Evolution

Exploring the crack evolution process of concrete materials when they are damaged by compression can effectively detect and provide feedback on the actual damage process of concrete. Because the elastic wave is similar to the seismic wave in the damage of concrete materials, the b-value analysis in the seismic field is cited to analyze the crack development process when the concrete is damaged under compression. The maximum likelihood estimation method (MLE) [41] is selected for the b-value calculation, and the formula is shown in (1).

(1) $$b=\frac{n\mathrm{lg}e}{\sum_{1}^{n}\mathrm{lg}A_{i}-n\mathrm{lg}A_{\min}}$$

where *A* represents the amplitude of the AE signal, *A*_min_ represents the minimum amplitude of AE at each time, *n* is the number of event points at each time period, and *e* is the natural constant.

**Figure 4:**
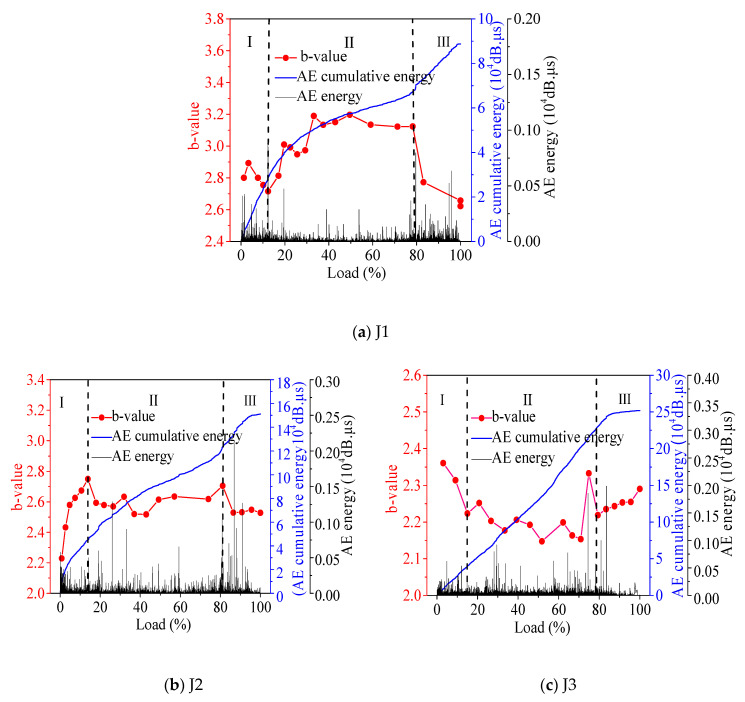

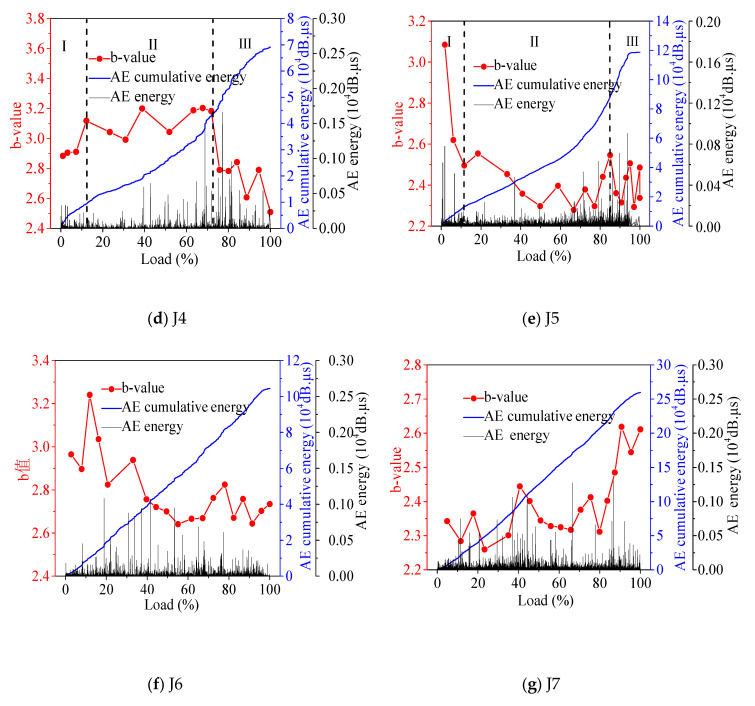
Concrete damage process.

According to the variation law of AE energy and b-value, along with load grade, the damage and destruction process of concrete is divided into three stages. The stage division is shown in Figure 4.

Phase I is the initial damage stage of the material. In this stage, the growth of microcracks and micropores is relatively small. The influence of the material density and friction between the surface of the test piece and the pressure bearing iron plate of the press have a sharp increase in AE energy. In Phase Ⅱ, the internal damage stage of the material develops steadily, and the concrete microcracks develop slowly, in which AE value size b fluctuates smoothly, and the AE energy surge is not obvious. Phase Ⅲ is the material internal damage stage. In this stage, without the fiber doping ratio of concrete, the AE b-value is mainly a downward trend, and the internal microcracks of the specimen quickly become macrocracks. In the concrete proportioning of basalt fiber, b-value or volatility will increase. This is because fiber fracture and the fracture of microcracks occur simultaneously, thus increasing the proportion of microcracks and causing a change in b-value. In this phase, the AE energy appears to surge. The specimen internally releases a huge amount of strain energy until the specimen loses effectiveness and is destroyed.

When the b-value appears at an inflection point with obvious rise or fall, it means there has been a transformation of concrete in the damage stage. In the mix ratio of non-fiber concrete, the initial defect is large, the number of microcracks is in the majority, and the b-value tends to increase as a whole. In the proportion of concrete mixed with fiber, the fiber reduces the initial defects of the concrete, there are fewer microcracks, and the variation rule of b-value tends to decrease.

It needs to be pointed out here that in the concrete mix ratio of J6 and J7, there were many original defects in the recycled coarse aggregate, which continued to be damaged in the whole loading process and generate AE signals. The AE energy in this concrete changed without obvious regularity, so the concrete could not be divided into stages for analysis.

**Figure 5:**
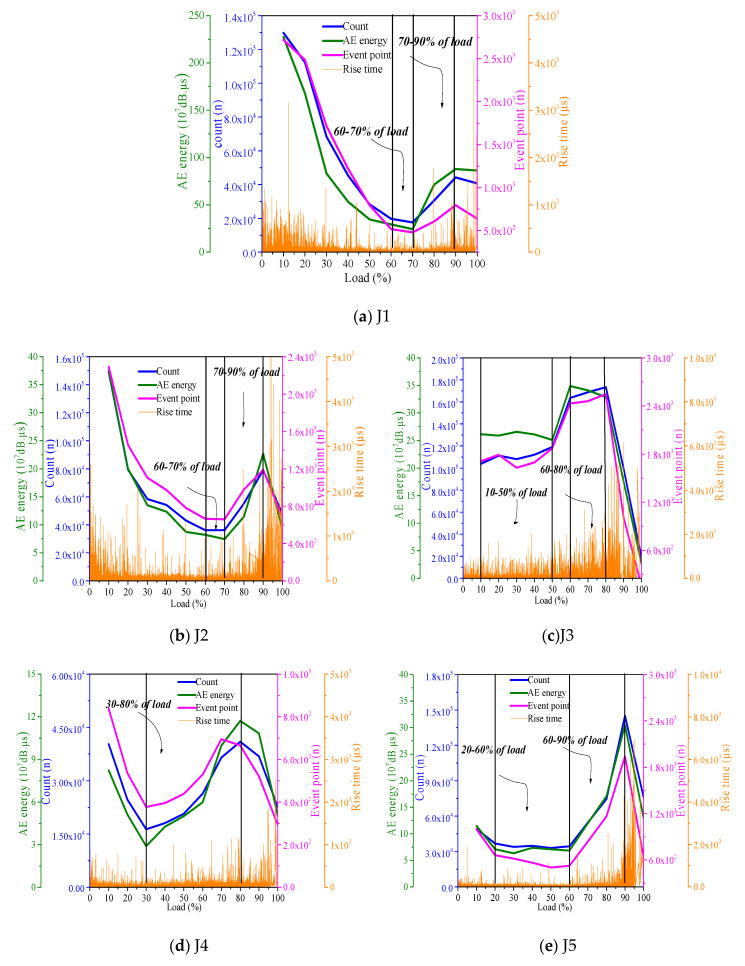

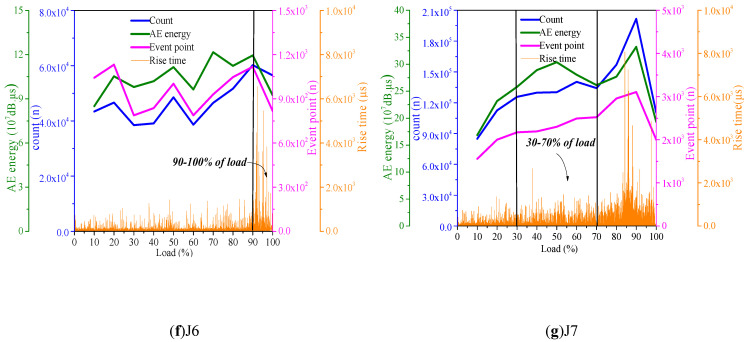
Analysis of AE activity and crack development rate of concrete.

According to Figure 5, the regularity of J1 and J2 is similar.

When the load level is 10–60%, the growth rate of the count, AE energy, and number of event points decrease, the rise time decreases, the AE activity gradually decreases from the initial maximum, and the microcrack growth rate slows down. When the load level is 60–70%, the increase of count, AE energy, and number of event points is relatively stable, AE activity is stable, and microcrack expansion is stable. When the load level is 70–90%, the increase of count, AE energy, and number of event points is rapid, and the crack rapidly expands. When the load level is 90–100%, the increase of count, AE energy, and number of event points is low; when the rise time increases, the microcracks gather to form macrocracks, the AE activity decreases, and at this time, the concrete fails. In J3, when the load level is 10–50% and 60–80%, the count, AE energy, number of event points, and rise time increase evenly, the AE activity is stable, and the microcracks expand steadily. When the load level is 50–60%, the count, AE energy, and number of event points increase rapidly, the AE phenomenon is active, and the microcracks expand rapidly. When the load level is 80–100%, the increase rate of the count, AE energy, and number of event points decreases, the AE activity decreases, and concrete damage occurs. Through the comparative analysis of J2 and J3, after the basalt fiber is added, the cracking of fully recycled concrete is stable in the early stage, but in the late damage process, the peak value rises earlier, and the failure of concrete occurs earlier.

In J4, the load level of the count, AE energy, number of event points, and increasing speed of rise time is 30–80%. At this stage, the microcracks appear quickly, and the AE activity is enhanced. The load grades of count, AE energy, number of event points, and rise time decrease are 10–30% and 80–100%. In this stage, the growth rate of microcracks decreases, and the AE activity decreases. In the 80–100% stage, microcracks gradually gather to form macrocracks, and the concrete is damaged. The change rule of J5 and J3 is similar, but the load levels of ring count, AE energy, and the number of event points that increase steadily, increase faster, and slow down are 20–60%, 80–90%, 10–20% and 90–100%, respectively. According to the comparative analysis of J4 and J5, doping basalt fiber reduces the AE activity of the early concrete under compression, but in the later failure stage, the fiber failure causes the AE phenomenon to be active and delays the rise time of the peak value, which inhibits the damage and failure of the recycled fine aggregate concrete.

During the whole process of J6 compression, the count, AE energy, and the number of event points increase steadily, the load level is 90–100%, and the rise time increases; J7 has the same development trend as J3 and J5, and the load levels of the count, AE energy, and the number of event points are 30–70%, 10–30%, 70–90%, and 90–100%, respectively. Compared with J6, J7 lags behind the rise time of the peak value, which shows that fiber plays a role in delaying the failure of recycled coarse aggregate concrete.

### 3.4. Failure State of Concrete

#### 3.4.1. Principle of k-Means Clustering

The k-means clustering algorithm is the most classical clustering method. The basic idea of the algorithm is to cluster with k points in space as the center and classify the objects closest to them. Through the iterative method, the values of each clustering center are updated successively until the best clustering results are obtained. Suppose the sample data are *n* variables *X*_1_*~X_n_*, and the *n* variables are divided into *X*_1_*~X_K_* class. Let *N_i_* be the number of variables in class *i X_i_*, and *m_i_* be the mean of these variables. The distance formula adopts European distance, and the specific operation is as follows:

1. Randomly and evenly select k observation samples as the initial clustering center *m*_1_*~m_k_*;
2. If:
  (2) $$d(x_{1},m_{p})\leq d(x_{j},m_{i}),1\leq p\leq k,i=1.2,\ldots ,k$$
  then *x* is divided into class *P*.
3. Recalculate each cluster center:
  (3) $$m_{i}=\frac{1}{N}\sum_{x\in x_{i}}x,i=1,2,\ldots ,k$$
4. Repeat steps (2) and (3) until the *m_i_* no longer changes.

#### 3.4.2. Optimal Number of Clusters

**Figure 6:**
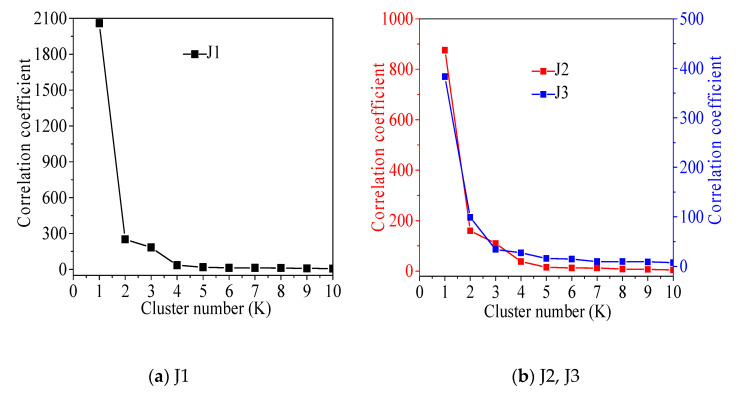

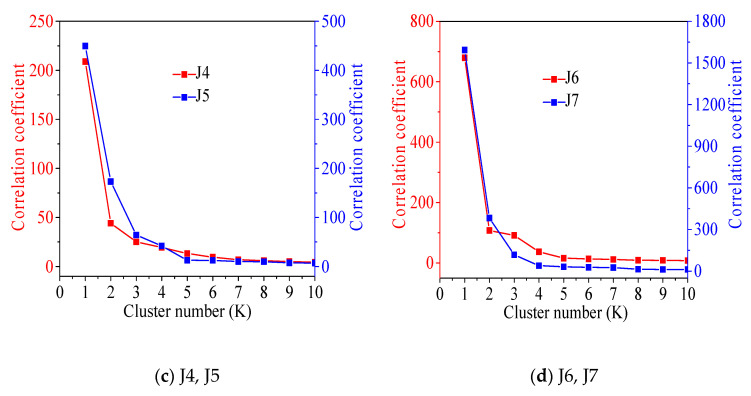
The optimal clustering number is determined.

The amplitude and peak frequency of AE are used as the parameters of cluster analysis, and the system clustering method in IBM SPSS Statistics 24 is adopted to obtain the optimal clustering number according to the relationship between the clustering correlation coefficient and the number of categories. The first obvious inflection point appears, and the curve gradually flattens, so this point can be selected as the optimal clustering number. According to Figure 6, the optimal number of clusters in J1, J2, J4, and J6 concrete is 2; the optimal number of clusters in J3, J5 and J7 concrete is 3.

#### 3.4.3. Analysis of Clustering Results

According to the relevant literature [36,37,38,39], damage states of J1, J2, J4, and J6 are divided into two categories, matrix cracking and interface debonding, and damage states of J3, J5 and J7 are divided into matrix cracking, interfacial debonding, and fiber cracking. The clustering principle of the concrete damage state is shown in Figure 6.

According to the results of cluster analysis, matrix cracking is wide value low peak frequency, interface debonding is medium value medium peak frequency, and fiber cracking is narrow value high peak frequency. Because there are many microcracks and holes on the surface of recycled aggregate, there are many uneven impurities along with old mortar with low strength on the surface. Thus, the recycled aggregate is prone to serious damage under pressure, resulting in high-frequency AE event points. Therefore, the peak frequency of recycled aggregate concrete is higher than that of natural concrete J1.

In the mixed proportion of recycled concrete, basalt fiber leads with the lower peak frequency of concrete matrix cracking and interface debonding because fiber improves the aggregate interface and cement mortar matrix and reduces the occurrence of high-frequency event points. When the concrete is going to be damaged, the fiber cracking effect is obvious, resulting in high-frequency event points, so the peak frequency is high. The peak frequency of recycled concrete can be the boundary point between fiber cracking and interface debonding. According to the clustering results, it can be seen that the limit peak frequency of fiber cracking and interface debonding in the recycled aggregate concrete is 250–300 kHz; the limit peak frequency of fiber cracking and interface debonding in the recycled fine aggregate concrete is 180–200 kHz; the peak frequency of 400 kHz in the recycled coarse aggregate concrete is the boundary point of interface debonding and fiber cracking.

By comparing the damage state of concrete caused by the coarse aggregate, fine aggregate, and basalt fiber, the main influencing factors of concrete compression damaged state are explored.

**Figure 7:**
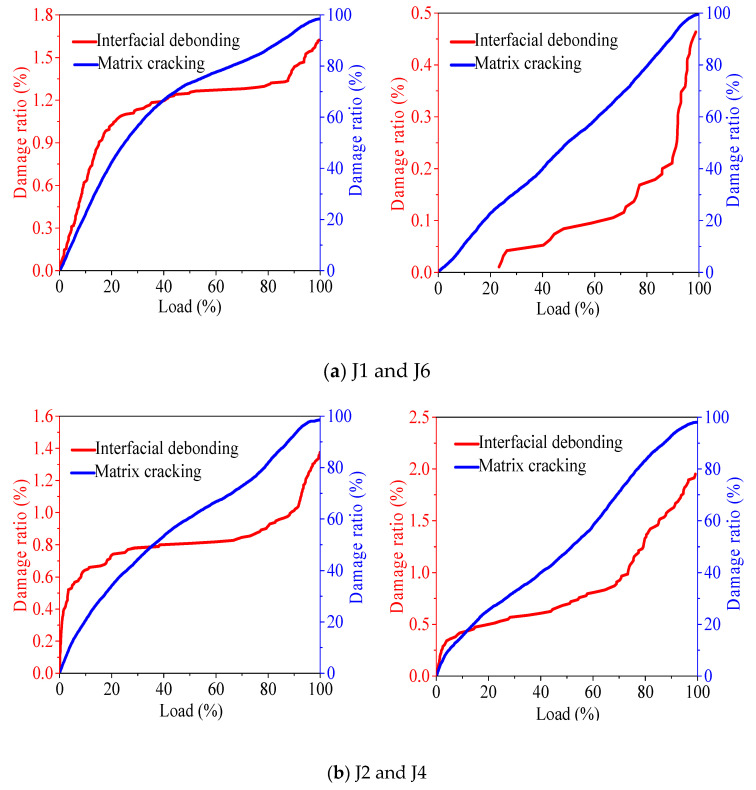
The influence of coarse aggregate on the failure state of concrete.

Figure 7 shows the effect of coarse aggregate on the damage state of concrete. According to Figure 7, matrix cracking is the most important damage state in concrete, and interface debonding is rare. In the proportion of natural concrete, when recycled coarse aggregate is used to replace the natural coarse aggregate, the interface debonding ratio decreases, and the matrix failure ratio increases. The reasons are as follows. First, the recycled coarse aggregate does not form a good interface with the natural river sand, and the interface is prone to compression failure, which reduces the interface debonding damage during the whole failure process of concrete; Second, there are microcracks and cracks on the surface of the recycled coarse aggregate, which are more likely to be damaged under pressure, resulting in the reduction of the duration and effect of the interface debonding process, so the matrix bears more damage. In the mix proportion of fully recycled concrete, when natural coarse aggregate is used instead of recycled coarse aggregate, the interface debonding ratio will increase, which is mainly due to the good strength and physical characteristics of natural coarse aggregate, and the interface strength is mainly related to the strength of coarse aggregate. Therefore, when the concrete is compressed, the aggregate cracking time and the interface debonding ratio will increase.

**Figure 8:**
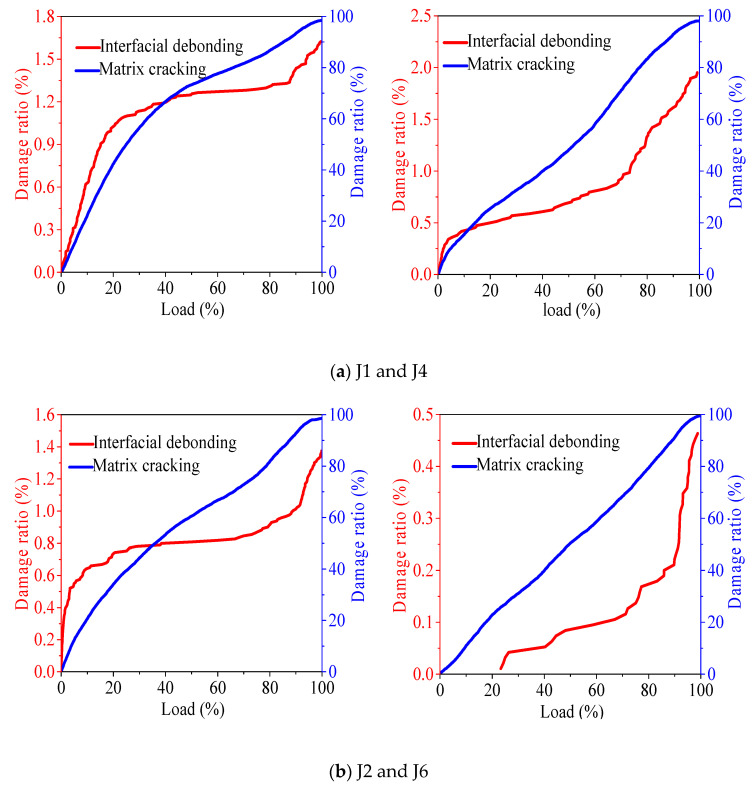
The influence of fine aggregate on the failure state of concrete.

Figure 8 shows the effect of fine aggregate on the damage state of concrete. As shown in Figure 8a, in natural concrete, recycled fine aggregate is used to replace all natural river sand, and the results show that the interface debonding damage increases during the whole failure process, while the matrix cracking damage decreases. This is because the strength of the mortar formed by recycled fine aggregate and cement is lower than that of the mortar formed by natural river sand and cement, and the compressive strength of the matrix is lower. Thus, the pressure bearing effect and the time of matrix cracking, along with the proportion of matrix cracking and destruction, are reduced, and the proportion of interface debonding and destruction is increased. Figure 8b shows the proportion of fully recycled concrete after using natural river sand instead of recycled fine aggregate, in which the proportion of matrix cracking and failure increases, and the interface debonding decreases. This is mainly because the recycled fine aggregate belongs to coarse sand, and the sand rate is higher than that of natural river sand. Recycled fine aggregate concrete that has been poured and formed is prone to water oozing, which makes the strength of the formed cement mortar lower than that of natural river sand and cement, and means the continuous bearing capacity is poor and is easier to crack. Therefore, the natural river sand replaces recycled fine aggregate to increase the proportion of concrete matrix cracking.

**Figure 9:**
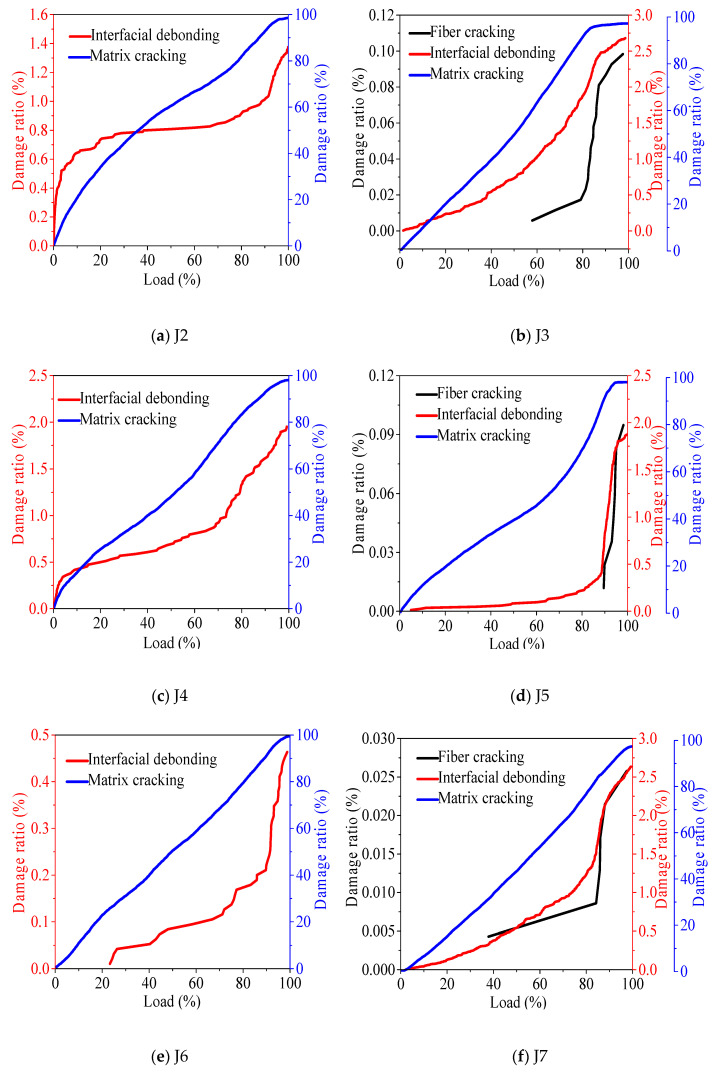
The influence of basalt fiber on the failure state of concrete.

Figure 9 shows the effect of basalt fiber on the damage state of recycled concrete. According to the analysis of (a) and (b) in Figure 9, after fully recycled concrete is doped with basalt fiber, the fiber optimizes the interface binding capacity of recycled aggregate, improves the bonding performance in recycled aggregate, makes the interface strength higher, and finally leads to the increase of interface debonding failure. When the load level is 60%, fiber cracking occurs, but the proportion in the overall failure of concrete is less than 0.11%. According to the analysis of (c) and (d) in Figure 9, after the recycled fine aggregate concrete is doped with fiber, the interface debonding failure ratio does not change significantly. When the load level is 90% left, the interface debonding failure ratio increases sharply, and the fiber cracking also starts from about 90% load, which indicates that the fiber cracking affects the interface debonding failure rate. In combination with (e) and (f) in Figure 9, after the recycled coarse aggregate concrete is mixed with basalt, the interface debonding ratio increases. When the load level is 40%, the fiber cracking begins to play a role; when the load level is 85%, the fiber cracking rate increases, and the interface debonding rate also increases, indicating that the fiber cracking rate affects the interface debonding failure rate.

### 3.5. Concrete Failure Mode

The failure modes of concrete under compression include tensile failure and shear failure. The correlation between *RA* and *AF* in the AE characteristic parameters is related to the failure type of concrete materials, which can effectively identify the failure type of concrete materials.

*RA* is defined as the ratio of rising time to amplitude in μs/V, as shown in Formula (2), and *AF* is defined as the ratio of ringing count to duration in kHz, as shown in Formula (3). There are differences between *RA* and *AF* values in shear damage and tensile damage. In tensile damage mode, due to the release of energy, the related *AF* values will be higher. Conversely, *RA* values associated with shear damage patterns are higher due to longer rise time and duration. The unit of amplitude in the original AE data of this test is (dB), which needs to be converted to (V). The conversion formula is shown in Formula (4).

(4) RA=Risetime/Amplitude

(5) AF=Counts/Duration

(6) $$dB_{AE}=20\log\left(V_{AE}/1\mu V\right)\left(\mathrm{mv}\right)$$

**Figure 10:**
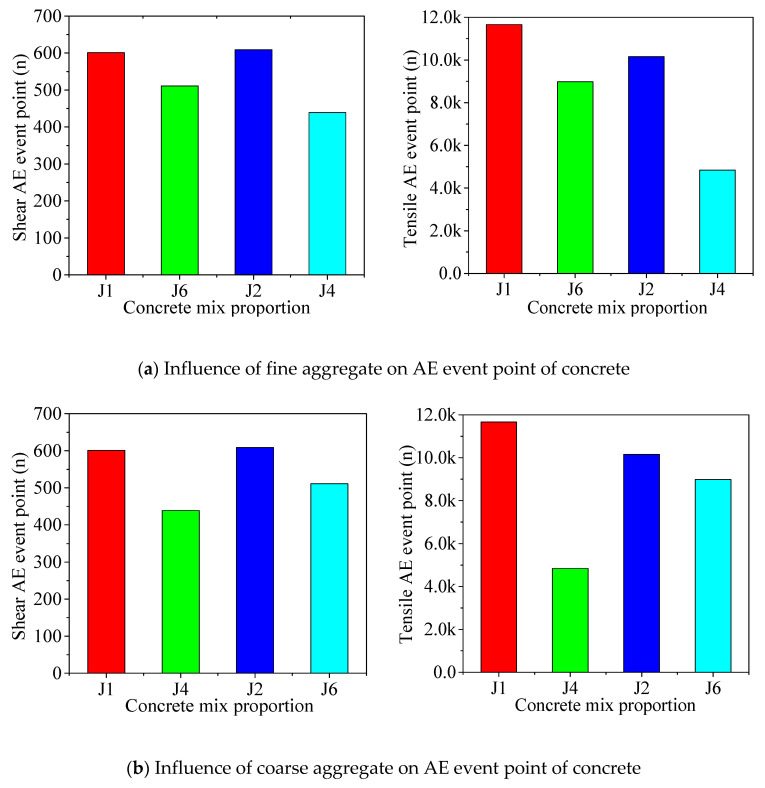
Influence of coarse and fine aggregate on AE event point of concrete.

In the data analysis, the k-means clustering analysis method in IBM SPSS Statistics 24 is used to divide *RA*-*AF* values into two categories. The concrete failure activity is closely related to the AE event point. In Table 7, the number of AE events of tensile and shear failure of concrete is counted, and the influence of fiber on AE tensile and shear event points is analyzed. Figure 10 shows the influence of coarse and fine aggregate on the AE event point of concrete.

The number of AE event points of natural concrete is more than that of recycled concrete because, in natural concrete, there are fewer initial defects and fewer micro cracks on the surface of natural aggregate than that on the surface of recycled aggregate; moreover, the aggregate strength is higher. As such, the interface strength of aggregate mortar is greater than recycled aggregate concrete, which eventually leads to the increase of concrete compression duration and more AE event points. After doping basalt fiber, the shear tensile failure event points in recycled concrete increase obviously, because fiber delays the transverse and longitudinal cracking of the concrete, and more AE event points are needed to counteract the effect of fiber on concrete cracking. At the same time, fiber cracking is also accompanied by the occurrence of event points, which eventually leads to an increase of AE cumulative event points.

As shown in Figure 10, whether recycled coarse aggregate is used to replace the natural coarse aggregate completely or recycled fine aggregate is used to replace the natural river sand completely, the tensile shear AE event points in the concrete are reduced because the strength of the recycled aggregate is lower than the natural aggregate, and the compressive performance of the recycled aggregate concrete is worse than the natural concrete, resulting in the decrease of the compression duration of the concrete. Consequently, the number of event points will be reduced. In fully recycled concrete, whether natural coarse aggregate is used to replace recycled coarse aggregate or natural river sand is used to replace recycled fine aggregate, the AE tensile shear event points in concrete decrease because of the collocation between the aggregates. The recycled aggregates have good material uniformity and can more easily form the high-strength concrete skeleton structure. Thus, the longer the compression time, the more AE event points are generated.

**Figure 11:**
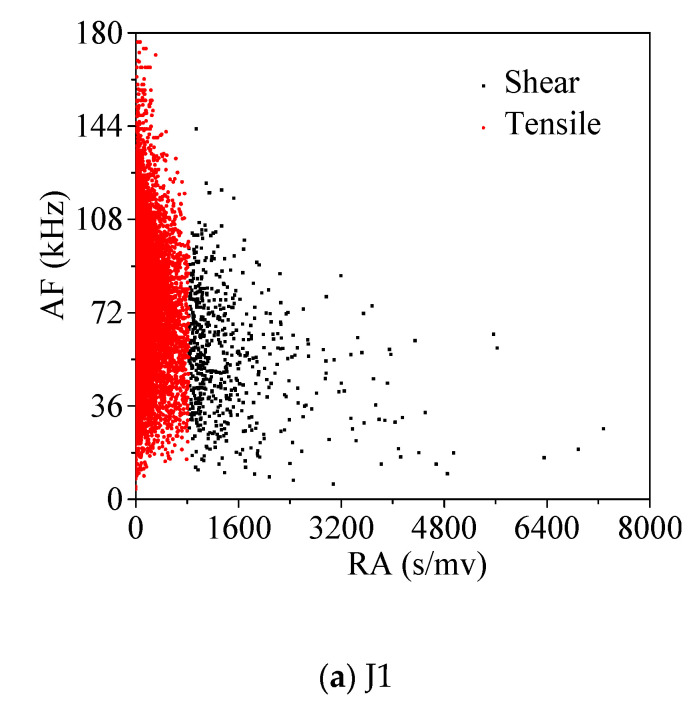

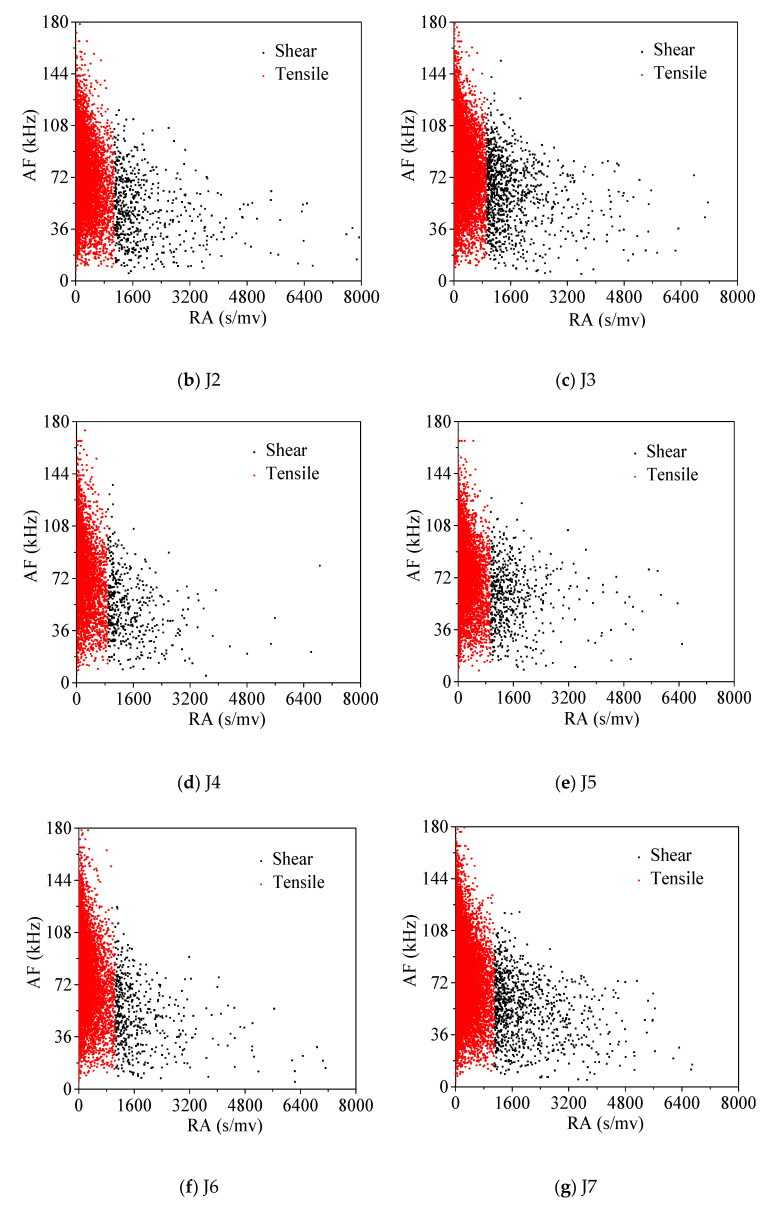
Concrete SPSS shear-tensile failure calculation results.

In this paper, the RA-AF value of concrete is divided into two clusters by cluster analysis method. Figure 11 shows the sheer tensile failure schematic diagram of concrete, in which the red part represents the tensile damage and the black part represents the shear damage.

**Figure 12:**
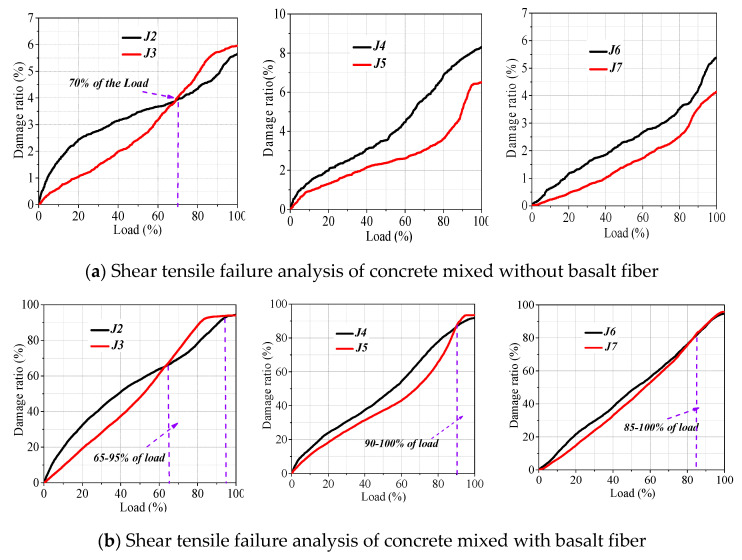
Shear tensile failure analysis of concrete mixed with and without fiber.

Figure 12 shows the influence of basalt fiber on the tensile shear damage of recycled concrete. In the fully recycled concrete, in the early stage of compression, the shear tensile failure ratio of concrete mixed with basalt fiber decreases. When the load level reaches 65–95% and 70%, the ratio of tensile failure to shear failure increases. This is mainly because the fiber inhibits transverse and vertical cracking in the early stage of concrete, which results in the decrease of shear tensile failure ratio in the early stage of compression. Combined with the analysis of the compressive strength of concrete, the strength becomes lower after fiber doping, which is more prone to transverse and longitudinal cracking in the failure stage. Meanwhile, fiber fracture also improves the tensile failure activity of recycled concrete. Finally, the recycled concrete makes the shear tensile failure ratio increase. With the increase of load level, the proportion of shear failure in the recycled coarse/fine aggregate ratio decreases with the addition of basalt fiber. When the load level reaches 90% and 85%, respectively, the ratio of tensile failure of concrete with fiber begins to exceed that of concrete without fiber. This is because after fiber doping, the transverse and longitudinal cracking of concrete is restrained in the early damage stage, and the tensile shear damage is reduced. However, when approaching the damage stage of concrete, the fiber cracking is also accompanied by tensile damage, which improves the tensile damage activity of concrete.

## 4. Conclusions

In this paper, the AE characteristic parameters of recycled concrete in the process of compression were collected by the test method of monitoring concrete compression in the whole process of AE, and the influence factors of concrete compressive strength were explored by taking aggregate mix mode and fiber as research variables. The AE b-value method was used to analyze the crack evolution process of recycled concrete in the process of compression, and AE cluster analysis was used to analyze the main damage state of recycled concrete in the process of compression. Finally, the AE RA-AF correlation method was used to explore the damage mode of concrete in the process of damage. The following conclusions were drawn:

(1) The compressive strength of concrete was greatly affected by the method of aggregate collocation. The strength of J7 was the highest, and that of J4 was the lowest. When the concrete reached the curing age, the strength of full recycled concrete decreased by 6.7%, and the strength of recycled fine aggregate concrete and recycled aggregate concrete increased by 4.5% and 5.0%, respectively. The best ratios of strength grade were J1, J2, and J7, all of which were over 62 MPa.
(2) Based on the b-value analysis of acoustic emission, the transformation process of concrete microcracks and macrocracks was explored from the mechanism. At the same time, combined with the analysis of AE energy, the concrete damage was divided into three stages: the initial damage of the material, the stable development of internal damage of the material, and the damage to the internal damage of the material. Basalt fiber could effectively inhibit the crack growth rate in the early stage of recycled concrete. In the later stage of failure, fiber led to the early failure of recycled concrete but delayed the failure of recycled fine/coarse aggregate concrete.
(3) There were two damage states of matrix cracking and interface debonding in common natural/recycled concrete and three damage states of matrix cracking, interface debonding, and fiber cracking in fiber recycled concrete. Matrix cracking, the main damage state of concrete, was wide value low peak frequency, the interface debonding was medium value medium peak frequency, and the fiber cracking was narrow value high peak frequency.
(4) In fully recycled/recycled fine/recycled coarse/aggregate concrete, the peak frequencies of the boundary point between fiber cracking and interface debonding were 250~300, 180~200, and 400 kHz, respectively, and the nodes of fiber cracking were 65%, 90%, and 85% of the load grade, respectively. The better the mortar strength was, the larger the ratio of matrix cracking damage was. The higher the amount of natural coarse aggregate, the greater the ratio of interface damage. The greater the rate of fiber cracking was, the greater the rate of interface debonding damage of recycled coarse and fine aggregate concrete.
(5) When the load levels were 65–95%, 90–100%, and 85–100%, basalt fiber improved the tensile failure activity of recycled/recycled fine aggregate/recycled coarse aggregate concrete. The addition of basalt fiber reduced the shear failure of recycled fine and coarse aggregate concrete, and when the load grade reached 70%, the shear failure of fully recycled concrete was enhanced by basalt fiber.

## Figures and Tables

**Table 1:** Physical properties of cement.

Standard Consistency (%)	Fineness (%)	Setting Time (min)		Flexural Strength (MPa)		Compressive Strength (MPa)	
		Initial	Final	3 d	28 d	3 d	28 d
28.40	6.0	194	271	5.5	8.9	26	48.5

**Table 2:** Quality index of fly ash (%).

Quality Index	Fineness	Ignition Loss	Water Content	Water Demand Ratio	SO_3_
Type I	12	5	1	95	3
The measured	11	0.38	0.2	89	0.78

**Table 3:** Short cut basalt fiber performance index.

Fiber Diameter (μm)	Moisture Content (%)	Linear Density (tex)	Combustible Content (%)	Breaking Strength (N/tex)	Length (mm)
16.6	≤0.2	2409	0.85	0.41	18

**Table 4:** Concrete material scale (kg/m^3^).

Cement	Water	Fly Ash	Sand/Slag	Fresh/Recycled Coarse Aggregate				
				4.75–9.5	9.5–13.2	13.2–16	16–19	19–26.5
420	150	80	714	179.4	296.2	296.6	182.4	31.6

**Table 5:** Aggregate detail sheet (kg/m^3^).

No.	Water	Sand	Slag	Fresh Coarse Aggregate	Recycled Coarse Aggregate	Basalt Fibers	Water Reducing Agent
J1	150	714	—	986	—	—	18.89
J2	150	—	714	—	986	—	36.12
J3	150	—	714	—	986	2.4	36.95
J4	150	—	714	986	—	—	28.04
J5	150	—	714	986	—	2.4	28.87
J6	150	714	—	—	986	—	29.52
J7	150	714	—	—	986	2.4	31.01

**Table 6:** Analysis of concrete compressive strength (MPa).

No.	Coarse Aggregate				Fine Aggregate				Basalt Fiber					
	J1	J6	J2	J4	J1	J4	J2	J6	J2	J3	J4	J5	J6	J7
3 d	46.5	40.6	50.1	43.3	46.5	43.3	50.1	40.6	50.1	46.1	43.3	43.9	40.6	42.8
Ratio of strength increase	12.7%↓		13.6%↓		6.9%↓		18.9%↓		8%↓		1.9%↑		5.4%↑	
7 d	51.4	46.3	53.9	47.1	51.4	47.1	53.9	46.3	53.9	48.9	47.1	48.4	46.3	47.1
Ratio of strength increase	9.9%↓		12.6%↓		8.4%↓		14.1%↓		9.2%↓		2.7%↑		1.7%↑	
28 d	62.8	60.2	62.5	57.2	62.8	57.2	62.5	60.2	62.5	58.3	57.2	59.8	60.2	63.2
Ratio of strength increase	4.1%↓		8.5%↓		8.9%↓		3.6%↓		6.7%↓		4.5%↑		5.0%↑	

**Table 7:** Statistics of concrete AE event points.

No.	J1	J2	J3	J4	J5	J6	J7
Tensile	601	609	1029	439	549	511	964
Shear	11,663	10,156	16,252	4842	7891	8980	22,313
